# A quantitative study of China’s rehabilitation policies from the perspective of policy tools

**DOI:** 10.3389/fpubh.2025.1676931

**Published:** 2025-12-01

**Authors:** Xinyang Jiang, Li Luo

**Affiliations:** School of Public Health, Fudan University, Shanghai, China

**Keywords:** rehabilitation, China, policy tool, policy analysis, rehabilitation policy

## Abstract

**Purpose:**

Rehabilitation policies constitute a critical guiding framework for national and regional development. This study aims to use policy tools to analyze the specific content and structural characteristics of China’s rehabilitation-related policies from 2010 to 2023, and provide a new reference perspective for international rehabilitation policy research.

**Materials and methods:**

A three-dimensional analytical framework of “policy tool type–policy theme characteristic–stakeholder” was developed. Data were drawn from nation-level policies on rehabilitation medical services issued by the State Council of the People’s Republic of China and the National Health Commission. Based on established inclusion and exclusion criteria, 41 valid policy documents were selected. NVivo (release 14.23.1) was used for four-level coding, and reliability and validity tests were conducted. Policy texts were first quantified, followed by descriptive analysis.

**Results:**

A total of 298 codes were identified. Among policy tool types, environmental tools accounted for the highest proportion (58.05%), followed by supply-side tools (32.98%), while demand-side tools had the lowest proportion (9.06%). From the stakeholder perspective, provider organizations (42.95%) were the core target, followed by regulatory authorities (19.13%) and rehabilitation service recipients (19.80%). Clinical practitioners and the rehabilitation capital market each accounted for 9.06%, with most of the policies related to passive response-type policies.

**Conclusion:**

China’s current rehabilitation medical service policies have insufficient use of supply-side and demand-side tools, and uneven policy coverage among stakeholders. The structural application of policy tools should be optimized, particularly by strengthening the role of demand-side policy tools.

## 1 Introduction

Rehabilitation is defined as the multidisciplinary and coordinated application of interventions to reduce physiological, psychological, and social functional impairments in individuals with illnesses, injuries, or disabilities (1). It primarily focuses on improving patients’ functional abilities following successful clinical treatment to support their continued daily living. Empirical evidence shows that rehabilitation is significantly cost-effective (2), enhances quality of life (3, 4), shortens hospital length of stay (5), lowers readmission rates (6, 7), and enhances occupational reintegration efficacy (8).

With the acceleration of global population aging and rapid development of medical technology, the demand for rehabilitation has increased rapidly in many countries and regions (9, 10). In response, developed countries in Europe and America have introduced numerous policies and legislative measures to promote the development of rehabilitation services (11, 12) and meet the growing needs of the population requiring rehabilitation services. As a developing country, China faces more complex challenges in this field. Between 1990 and 2019, the number of individuals requiring rehabilitation services in China increased from 268 million to 460 million, reflecting a 71.6% increase, ranking among the highest globally (13).

Although rehabilitation plays an increasingly important role in addressing health governance challenges (14), international research has primarily focused on rehabilitation practices (15, 16), technologies (17, 18), and outcomes (19, 20). Most rehabilitation policy analyses have examined policy implementation (21) or specific disease categories (22), lacking comprehensive analysis from a holistic perspective and systematic examination of policy structures. Policy tools, defined as the mechanisms through which governments formulate, evaluate, and implement policy alternatives, constitute a central domain in policy science (23) and serve as a critical analytical framework in public policy research (24). The selection and application of policy tools have a significantly influence on a government’s capacity to achieve its policy objectives (25). Each tool provides operational guidance for the implementation and regulation of government policies in a concise, actionable form (26).

In this study, we sought to bridge the current research gap by applying policy tools to examine existing rehabilitation medical service policies in China. We identified specific indicators for each analytical dimension of policy tools and explored the feasibility of cross-analysis between policy tools and stakeholders, thereby providing a new reference perspective for international rehabilitation policy research. It is important to note that the “quantitative analysis” in this study refers specifically to the process of converting non-numerical information such as policy texts into measurable numerical data, rather than the broader methodological distinction between quantitative and qualitative research.

## 2 Materials and methods

### 2.1 Information sources

This study systematically retrieved policy documents on rehabilitation services from the State Council Policy Repository. The primary search terms were “rehabilitation,” “rehabilitation medical services,” “rehabilitation medicine,” and “rehabilitation medical system.” The search covered policy documents issued between October 1, 2010, and October 1, 2023, by the State Council, the General Office of the State Council, and ministerial bodies under the State Council, including the National Health Commission, Ministry of Civil Affairs, National Healthcare Security Administration. To ensure thematic relevance and methodological validity, the initially identified policy corpus was subjected to rigorous screening using pre-established eligibility criteria, encompassing both inclusion and exclusion conditions:

#### 2.1.1 Inclusion criteria

- Documents containing substantive provisions directly related to rehabilitation service delivery mechanisms.
- Nation-level tools (e.g., notices, decisions, and guidelines) issued through official State Council channels.

#### 2.1.2 Exclusion criteria

- Documents of low relevance, such as replies or inter-departmental forwarded letters.
- Documents that only mention keywords without providing substantive content.
- Duplicate documents.

Based on these criteria, a total of 41 policy documents met the eligibility requirements and were included in the Rehabilitation Medical Service Policy Database. Representative examples are provided in Table 1.

**Table 1:** Examples of policy documents related to rehabilitation services.

No.	Document	Source	Date
1	Notice on the Inclusion of Some Medical Rehabilitation Programs in Basic Medical Care Coverage	Ministry of Health, PRC Ministry of Human Resources and Social Security, PRC Ministry of Civil Affairs, PRC	2010-09-06
2	Ministry of Health Pilot Program for Establishing and Improving the Rehabilitation Medical Service System	Ministry of Finance, PRC China Disabled Persons’ Federation	2010-08-30
3	Circular of the Ministry of Health on the Issuance of the Guiding Opinions on Rehabilitation Medical Care in the Twelfth Five-Year Plan Period	Ministry of Health, PRC	2012-02-29
4	Opinions of the General Office of the State Council on Promoting the High-Quality Development of Public Hospitals	General Office of the State Council, PRC	2021-05-14
5	Opinions on Further Improving the Medical and Healthcare Service System	CPC Central Committee General Office General Office of the State Council, PRC	2023-03-23

### 2.2 Textual coding

The coding of samples began with manual intensive reading of policy texts, followed by content consolidation and hierarchical coding of the texts using NVivo release 14.23.1 (38) software. The coding adopts a four-level structure: “policy number - chapter number - section number - text content.” Subsequently, frequency statistical analysis and comparative analysis were conducted on the policy samples to summarize the composition of rehabilitation medical service policy tools in mainland China and propose optimization suggestions. As shown in Table 2, documents were numbered in chronological order of issuance, with the example document assigned number 8; its third chapter is numbered 3; the tenth subsection of Chapter 3 is numbered 10; each sentence containing specific descriptions within the subsection is assigned an individual code. In this subsection example, the first sentence is coded as 1, resulting in the final code 8–3–10-1. Only codes related to rehabilitation policies were included in the result analysis, while those not related to rehabilitation policies were excluded.

**Table 2:** Example of rehabilitation policy text coding.

Policy ID	Policy title	Chapter	Section	Policy content	Policy code	Tool type	Tool name	Stake-holder
8	Notice of the State Council on Issuing the “13th Five-Year” Plan for Health and Health	Chapter three: main tasks	Section (10) accelerate the development of the health industry	Encourage social forces to develop resource-scarce services that meet diverse needs, including pediatrics, psychiatry, geriatrics, long-term care, oral health care, rehabilitation, and hospice care.	8-3-10-1	Demand-side	Market development incentivization	Capital Market

### 2.3 Reliability and validity testing

To ensure the reliability of rehabilitation policy coding, two coders independently conducted simultaneous coding. Before coding, both coders engaged in detailed discussions to reach consensus on coding categories, principles, and procedures. After completing their coding independently, the coders compared their results to generate two sets of coding outcomes. These were subsequently evaluated by experts who reviewed the consistency between the two sets, identified discrepancies, and facilitated revisions through group discussions. Ultimately, Group A identified 292 codes related to the research theme, while Group B selected 301 such codes. Among these, 286 codes were rated as consistent between the two groups. Following group discussions, the final set of valid codes was confirmed to be 298. The percentage of agreement for reliability stood at 93.16%, and the percentage of agreement for validity reached 96.46%. These results indicate that the coding outcomes of this study are valid.

### 2.4 Analytical framework construction

**Figure 1:**
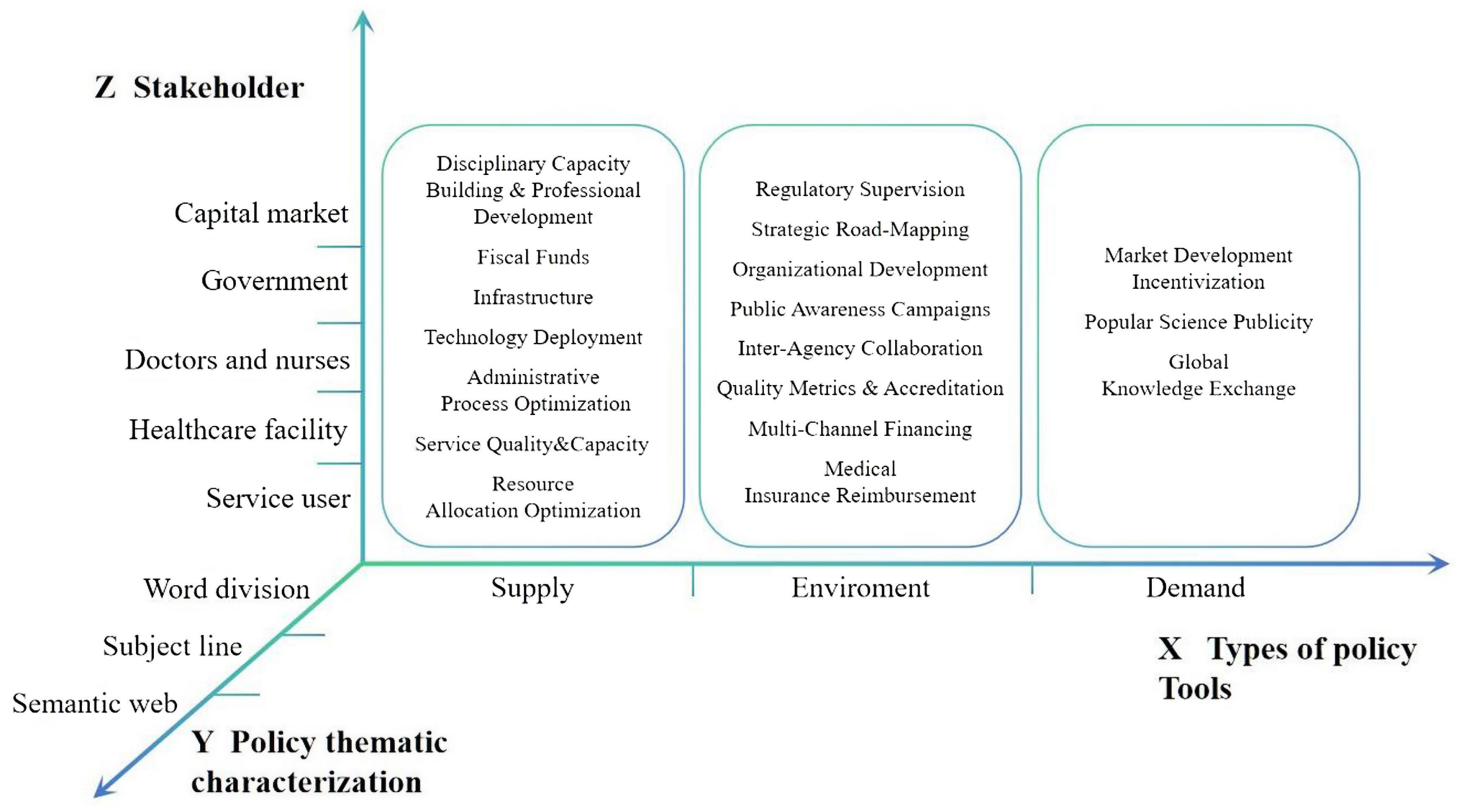
Analytical framework for China’s rehabilitation medical service policy documents.

The constructed policy analysis framework for rehabilitation medical services comprised three constitutive dimensions: types of policy tools, thematic characterization of policies, and stakeholders (Figure 1).

#### 2.4.1 X-dimension: types of policy tools

This analytical component adopts the tripartite taxonomy of policy tools conceptualized by Rothwell and Zegveld (27)—supply-side, environmental, and demand-side tools—to systematically examine rehabilitation medical service policy documents.

From an international perspective, this framework has been widely applied in policy analysis within fields such as healthcare and industrial development, boasting a well-established theoretical foundation and operational paradigm. The 2010–2023 period represents a critical phase in the transformation of China’s rehabilitation medical services from scattered exploration to systematic development. Policy interventions during this period need to simultaneously address three aspects: the establishment of an institutional framework (environment), the supply of resource factors (supply), and the stimulation of service demand (demand).

Supply-side tools refer to the direct supply and support provided by the government for activities related to the construction and development of rehabilitation medical services, focusing on addressing weaknesses and strengthening foundations. For instance, fiscal funds can include special subsidies for primary-level rehabilitation equipment and transfer payments to poverty-stricken areas. Environmental tools denote a series of measures adopted by the government to create a favorable development environment for the growth of rehabilitation medical services, emphasizing framework construction and collaboration promotion. A case in point is the joint promotion of the “Integration Initiative for Mental Health and Rehabilitation Services” by multiple ministries and commissions. Demand-side tools refer to the approaches employed by the government, such as popular science publicity, market development, and international exchanges, to enhance the public’s demand for rehabilitation medical services.

After subdividing the specific categories in the rehabilitation medical service policy texts, a total of 18 tool categories were identified. The specific categories, their definitions, and illustrative examples are presented in Table 3.

**Table 3:** Classification and conceptual analysis of policy tools.

Type	Name	Meaning	Specific Policy Text Examples
Supply-side	Fiscal funds	Governmental fiscal mechanisms to ensure the sustainable development of rehabilitation service delivery systems through targeted fiscal interventions across implementation phases, including direct financial provisioning and capital injections.	Local governments are encouraged to establish a stable funding input mechanism to ensure funds for infrastructure construction, equipment and facilities, staff salaries, training, and other operational expenses.
	Infrastructure	Infrastructure development for software and hardware related to rehabilitation services, including the expansion of rehabilitation-related medical institutions, increasing ward capacity and building space, enhancing medical treatment facilities, upgrading equipment, and improving staffing.	Strengthen the construction of continuous medical institutions for rehabilitation, geriatric care, long-term care, chronic disease management, and hospice care.
	Disciplinary capacity building & professional development	Strengthening the construction of rehabilitation disciplines, enhancing skill acquisition, and developing relevant professional talent teams.	Set up a special program for the training of Traditional Chinese Medicine (TCM) rehabilitation talents within the TCM characteristic talent training project.
	Administrative process optimization	Implementation of expedited review priorities for rehabilitation facility license, including streamlined approval protocols for rehabilitation hospital establishment and fast-tracking accreditation for social capital-funded rehabilitation clinics.	Priority in establishment approval shall be given to applicants for setting up group-based and chain-operated rehabilitation medical centers and care centers.
	Technology deployment	Improving the informational construction of rehabilitation services, promoting the transformation of rehabilitation-related scientific and technological achievements, and the application of appropriate technologies.	Promote the transformation of scientific and technological achievements and the application of appropriate technologies.
	Resource allocation optimization	Optimizing staffing and equipment allocation, clarifying the functional roles of medical institutions at all levels in the development of rehabilitation medicine, strengthening the guidance and support provided by higher-level medical institutions to lower-level ones, promoting the implementation of hierarchical diagnosis and treatment, and establishing medical associations.	Tertiary hospitals and medical institutions for chronic diseases such as rehabilitation and care institutions may provide cross-grid services; resources including inspection and testing, disinfection supply, and special clinical specialty technologies may be shared within the region.
	Service quality & capacity	Enhancing the service capacity and standardized management level of rehabilitation medicine, improving the quality of rehabilitation medical services, and expanding the service population and scope.	Strengthen primary-level services, address weaknesses, and improve medical service capabilities in urgent fields such as rehabilitation and care.
Environmental	Strategic road-mapping	The overall plan, specific objectives, major tasks, and general guiding principles for the future development of rehabilitation medical services, formulated based on the current state of these services.	Regions with conditions are encouraged to include basic rehabilitation services in the scope of personalized service contracts.
	Organizational development	Strengthen the leadership of Party organizations, implement relevant responsibilities, and enhance awareness.	Strengthen organizational leadership. Establish a working mechanism led by the Party committee, undertaken by the government, collaborated by relevant departments, and participated in by the society.
	Regulatory supervision	The orderly advancement of rehabilitation medical services is ensured by formulating and refining pertinent laws, regulations, and departmental rules, as well as by reinforcing the supervision of medical practice and implementing other mandatory measures.	Establish a recording and supervision system for community-based rehabilitation services for mental disorders.
	Quality metrics & accreditation	Standardized rehabilitation diagnosis and treatment techniques for common diseases, clinical pathways, and quality assessment criteria for rehabilitation therapy.	Conduct research on TCM rehabilitation protocols and technical standards.
	Multi-channel financing	Strengthening funding support, increasing subsidy levels, diversifying funding sources, and implementing incentive policies such as fee reductions and exemptions.	Local governments shall provide support to institutions offering rehabilitation and care services in communities in accordance with the law, including tax and fee reductions, financial support, and preferential prices for water, electricity, gas, and heating.
	Inter-agency collaboration	Strengthening communication, coordination, and cooperation among administrative departments, as well as collaboration between different departments and the rehabilitation medicine department.	Encourage geriatric care facilities to establish close connections and collaborative mechanisms with neighboring continuous medical institutions such as rehabilitation hospitals (rehabilitation medical centers), nursing homes (care centers), and hospice care centers.
	Public awareness campaigns	Strengthening publicity efforts regarding the basic principles, main tasks, and policy measures related to the pilot initiative of establishing and improving the rehabilitation medical service system.	All regions shall attach importance to and strengthen the publicity of rehabilitation medical service work.
	Medical insurance reimbursement	Expanding the scope of reimbursement under basic medical insurance and industrial injury insurance, increasing the reimbursement rate for rehabilitation-related services, and improving the payment method of medical insurance.	For diseases requiring long-term hospitalization with relatively stable daily costs, such as mental illness, hospice care, and medical rehabilitation, a per diem payment method may be adopted.
Demand-side	Popular science publicity	Utilizing various forms for comprehensive and multi-dimensional science publicity and education on disease rehabilitation knowledge for the population.	Vigorously publicize rehabilitation concepts, knowledge, and technologies to popularize and enhance the public’s awareness and attention to rehabilitation.
	Market development incentivization	Encouraging the participation of social capital and providing support and guidance for its entry into the field of rehabilitation medical services, thereby accelerating the development of rehabilitation-related industries.	Support social forces in providing services for the older adults, including day care, meal assistance, cleaning assistance, and rehabilitation care.
	Global knowledge exchange	Engaging in academic exchanges and collaboration in the field of rehabilitation with relevant international organizations and countries, to promote China’s experience in rehabilitation medicine and learn from the expertise and advanced technologies of other nations.	Actively create a sound international environment for the development of undertakings for persons with disabilities.

#### 2.4.2 Y-dimension: thematic policy characterization

Thematic policy characterization effectively reflects the prioritization logic of policy-promulgating entities during the policymaking process. Based on established analytical frameworks (28), thematic characterization in this study was deconstructed into three methodological components, including lexical segmentation, thematic keyword extraction, and semantic network analysis. A term frequency analysis was conducted using ROST CM 6.0 (developed by Wuhan University’s Big Data Research Team) on the 41 rehabilitation service policy documents to identify high-frequency keywords, construct a co-word matrix. Based on this co-word matrix, Gephi 0.10 was used to build a semantic network (29). The co-occurrence matrix algorithm was applied to map the lexical relationship patterns and reveal potential policy focus areas. This hierarchical analytical approach enables multilevel interpretation of policy texts, from surface-level lexical patterns to deeper semantic configurations.

#### 2.4.3 Z-dimension: stakeholder

Policy implementation requires collaborative engagement among multiple stakeholders to achieve strategic objectives (30). Stakeholder theory posits that organizational success depends on effectively managing relationships with key groups, including clients, employees, suppliers, communities, funders, and other entities influencing goal attainment (31). Since the 1990s, this framework has demonstrated strong applicability in health-sector research (32).

In alignment with rehabilitation policy characteristics, this study identified five core stakeholder categories as the Z dimension: regulatory authorities (government agencies), provider organizations (healthcare institutions), clinical practitioners (medical professionals), service recipients (patients), and rehabilitation capital markets (including equipment manufacturers and suppliers).

## 3 Results

### 3.1 X-dimension analytical findings

Analysis of the 41 policy documents yielded 298 coded segments. Frequency distribution across policy tool categories revealed significant disparities in their deployment (Table 4). Environmental tools dominated policy implementation (58.05%), followed by supply-side (32.98%) and demand-side tools (9.06%).

**Table 4:** Policy tool categorical distribution.

Type	Name	Quantity	%
Supply-side	Fiscal funds	2	0.67
	Infrastructure	6	2.01
	Disciplinary capacity building & professional development	26	8.72
	Administrative process optimization	2	0.67
	Health information technology deployment	7	2.35
	Resource allocation optimization	29	9.73
	Service quality & capacity	26	8.72
		98	32.89
Environment	Strategic road-mapping	101	33.89
	Organizational development	16	5.37
	Regulatory supervision	6	2.01
	Quality metrics & accreditation	5	1.68
	Multi-channel financing	3	1.01
	Inter-agency collaboration	25	8.39
	Public awareness campaigns	5	1.68
	Medical insurance reimbursement	12	4.03
		173	58.05
Demand-side	Popular science publicity	4	1.34
	Market development incentivization	21	7.05
	Global knowledge exchange	2	0.67
		27	9.06
Total		298	100

Quantitative analysis of environmental tool subtypes revealed significant disparities in their application. Further analysis of specific policy tool subcategories revealed that within environmental policy tools, strategic road-mapping constituted the largest proportion (33.89%), followed by inter-agency collaboration mechanisms (8.39%) and governance infrastructure enhancement (5.37%). Strategic road-mapping primarily involves the government’s formulation of developmental blueprints, specific objectives, and operational frameworks tailored to the current landscape rehabilitation service delivery. A representative example is the Precision Rehabilitation Service Initiative articulated in the National Health Commission’s policy documents: “Implement targeted rehabilitation interventions prioritizing children with disabilities and certified disabled individuals, achieving 80% coverage of essential rehabilitation services for the disabled population with rehabilitation needs.” Inter-agency collaboration mechanisms encompass both vertical administrative coordination (e.g., inter-ministerial task forces under State Council Directive 2021–19) and horizontal clinical integration models (e.g., multidisciplinary consultation systems linking rehabilitation departments to other clinical specialties).

Supply-side policy tools emphasize three strategic dimensions: rehabilitation resource allocation optimization (9.73%), service quality enhancement (8.72%), and disciplinary capacity building (8.72%). This finding indicates that the government prioritized equitable and rational allocation of rehabilitation medical resources, promoted service development through hierarchical diagnosis and treatment systems, and supported resource decentralization through the establishment of healthcare consortia. Additionally, it emphasized service quality and talent development to ensure these three strategic dimensions work synergistically.

Although demand-side interventions constituted the smallest proportion (9.06%) of policy tool categories, they reflect a notable policy emphasis on market development incentives (7.05%). This aligns with China’s decade-long policy trajectory of promoting social capital participation in rehabilitation services, as outlined by the State Council’s Guidelines on Social Capital Participation in Healthcare (2019). Implementing rehabilitation health literacy initiatives among the general population, alongside establishing international knowledge exchange frameworks, particularly with nations demonstrating advanced rehabilitation service systems, holds substantial potential for advancing the field. These endeavors align with the World Health Organization’s Rehabilitation 2030 Initiative (33).

### 3.2 Y-dimension analytical findings

#### 3.2.1 Lexical frequency analysis

The analytical protocol involved a systematic term frequency analysis of the 41 policy documents using ROST CM 6.0. Lexical segmentation and frequency quantification yielded a ranked list of the top 20 high-frequency keywords. The lexical segmentation outcomes are listed in Table 5.

**Table 5:** Top 20 high-frequency keywords in China’s rehabilitation medical service policies.

No.	Keywords	Frequency
1	Rehabilitation	1,038
2	Medical Care	629
3	Services	606
4	Institutions	278
5	Hospitals	180
6	Psychiatric	150
7	Management	149
8	Community	145
9	Strengthening	142
10	Health	141
11	Chinese Medicine	128
12	Health	128
13	Construction	124
14	Nursing	122
15	Capacity	115
16	Barriers	115
17	Professional	106
18	Treatment	105
19	Providing	104
20	Work	101

#### 3.2.2 Semantic network analysis of themes

**Figure 2:**
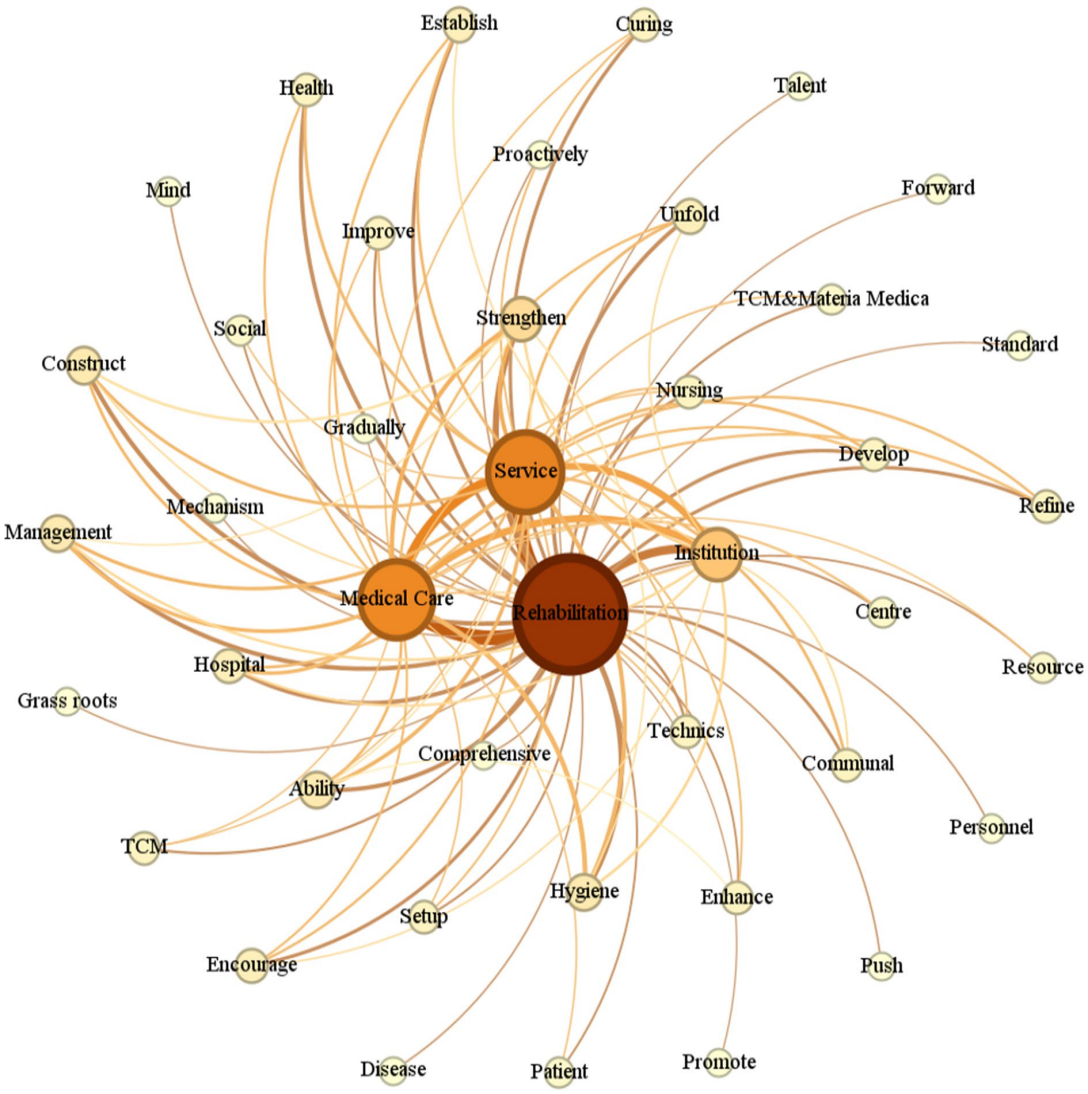
Semantic network map of keywords for China rehabilitation healthcare policy text. Key association pathways: rehabilitation and medical care, rehabilitation and institution, rehabilitation and strengthening, rehabilitation and service.

Building on the word frequency analysis, further semantic network analysis of theme words in the rehabilitation policy documents was conducted. As shown in Figure 2, strong similarities and correlations were observed among the theme words in the policy text. The core terms, service, medical care, rehabilitation, strengthening, and institution, highlight the policy emphasis on enhancing the provision and development of rehabilitation services in medical and health institutions. The secondary keywords included management, health, construction, establishment, development, improvement, and technology, indicating that China’s rehabilitation medical services remain in the developmental phase, with ongoing improvements in terms of both technological capacity and institutional infrastructure.

### 3.3 Z-dimension analytical findings

The analytical framework revealed that provider organizations were the predominant direct stakeholders in rehabilitation service delivery, accounting for 42.95% of policy interventions. Service recipients (patients) and regulatory authorities represented secondary stakeholder groups, while rehabilitation capital markets and clinical practitioners exhibited limited policy engagement (Table 6).

**Table 6:** Distribution of policies across stakeholders.

Stakeholder	Number of policies	%
Regulatory authorities	57	19.13
Provider organizations	128	42.95
Clinical practitioners	28	9.06
Service recipients	59	19.80
Rehabilitation capital market	26	9.06
Total	298	100

### 3.4 Results of cross-sectional analysis

Based on the single-dimensional analysis of rehabilitation medical service policies, a cross-dimensional analysis between specific policy tool categories and stakeholders was conducted. The two-dimensional quantitative results are shown in Table 7.

**Table 7:** Two-dimensional statistics of ‘specific categories–stakeholders’ in rehabilitation policy tools.

Type	Name	Service recipients	Clinical practitioners	Provider organizations	Regulatory authorities	Rehabilitation capital markets	Total
Supply-side	Fiscal funds	1	0	0	1	0	2
	Infrastructure	0	0	6	0	0	6
	Disciplinary capacity building & professional development	0	24	2	0	0	26
	Administrative process optimization	0	0	1	0	1	2
	health information technology deployment	2	0	3	2	0	7
	Resource allocation optimization	6	0	18	3	2	29
	Service quality & capacity	7	2	15	1	1	26
		16	26	45	7	4	98
Environment	Strategic road-mapping	27	0	60	13	1	101
	Organizational development	0	0	1	14	1	16
	Regulatory supervision	0	0	2	4	0	6
	Quality metrics & accreditation	1	0	1	3	0	5
	Multi-channel financing	0	0	2	0	1	3
	Inter-agency collaboration	2	1	14	7	1	25
	Public awareness campaigns	1	0	2	2	0	5
	Medical insurance reimbursement	6	1	0	5	0	12
		37	2	82	48	4	173
Demand-side	Popular science publicity	3	0	0	1	0	4
	Market development incentivization	3	0	0	0	18	21
	Global knowledge exchange	0	0	1	1	0	2
		6	0	1	2	18	27
Total		59	28	128	57	26	298

The X–Z analysis revealed that supply-side policy tools are mostly applied to healthcare institutions, healthcare workers, and patients. Additionally, environmental policy tools are mostly applied to healthcare institutions, government agencies, and patients, while demand-side tools are concentrated within the rehabilitation capital market, with minimal application to healthcare workers, healthcare institutions, and government agencies.

### 3.5 Results of temporal dimension analysis

To further reveal the dynamic development trajectory of China’s rehabilitation medical service policies, this study incorporated a temporal dimension and divided the policy samples into three phases based on China’s Five-Year Plans for National Economic and Social Development: the initial exploration phase (2010–2015, covering the 12th Five-Year Plan period and prior), the accelerated development phase (2016–2020, the 13th Five-Year Plan period), and the in-depth integration phase (2021–2023, the 14th Five-Year Plan period). The results are presented in Tables 8, 9.

**Table 8:** Changes in policy tools over time.

Time		2010–2015		2016–2020		2021–2023	
Type		Quantity	%	Quantity	%	Quantity	%
Supply-side	Fiscal funds	1	1.72	1	0.69	0	0.00
	Infrastructure	1	1.72	2	1.39	3	3.13
	Disciplinary capacity building & professional development	6	10.34	9	6.25	11	11.46
	Administrative process optimization	0	0.00	2	1.39	0	0.00
	Health information technology deployment	0	0.00	6	4.17	1	1.04
	Resource allocation optimization	10	17.24	10	6.94	9	9.38
	Service quality & capacity	3	5.17	10	6.94	13	13.54
		21	36.2	40	27.7	37	0.00
Environment	Strategic road-mapping	12	20.6	65	45.14	24	25.00
	Organizational development	2	3.45	5	3.47	9	9.38
	Regulatory supervision	1	1.72	3	2.08	2	2.08
	Quality metrics & accreditation	3	5.17	2	1.39	0	0.00
	Multi-channel financing	0	0.00	2	1.39	1	1.04
	Inter-agency collaboration	11	18.9	9	6.25	5	5.21
	Public awareness campaigns	2	3.45	0	0.00	3	3.13
	Medical insurance reimbursement	3	5.17	5	3.47	4	4.17
		34	58.62	91	63.1	48	0.00
Demand-side	Popular science publicity	0	0.00	0	0.00	4	4.17
	Market development incentivization	2	3.45	13	9.03	6	6.25
	Global knowledge exchange	1	1.72	0	0.00	1	1.04
		3	5.17	13	9.03	11	0.00
Total		58	100.00	144	100.00	96	100.0

**Table 9:** Temporal changes in stakeholders.

Time	2010–2015		2016–2020		2021–2023	
Type	Quantity	%	Quantity	%	Quantity	%
Service recipients	7	12.07	30	20.83	22	22.92
Clinical practitioners	5	8.62	11	7.64	12	12.50
Provider organizations	19	32.76	79	54.86	30	31.25
Regulatory authorities	23	39.66	9	6.25	25	26.04
Rehabilitation capital market	4	6.90	15	10.42	7	7.29
Total	58	100.00	144	100.00	96	100.00

In the initial exploration phase, environmental tools dominated. By frequently utilizing tools such as goal planning and inter-departmental collaboration, efforts were focused on establishing a basic institutional framework. From the perspective of stakeholders, policy focus was highly concentrated on government agencies and healthcare institutions, reflecting a path dependence on driving the initial development of rehabilitation initiatives through the internal administrative system.

During the accelerated development phase, the proportion of environmental tools further increased. Among supply-side tools, the use of information technology support and optimized administrative approval grew significantly, indicating a shift in resource allocation toward efficiency and innovation. The proportion of demand-side tools rose to 9.03%, with a focus on market development to encourage social capital participation in the rehabilitation sector. Among stakeholders, the proportion of policies targeting healthcare institutions jumped to 54.86%, making them the core of policy implementation. Concurrently, attention to patients (20.83%) and capital market participation (10.42%) increased, marking a shift in policy focus from institutional establishment to scale expansion.

In the in-depth integration phase, policy focus shifted from framework construction to connotative development, and the use of tools became more balanced. Among supply-side tools, service quality improvement, disciplinary development, and talent education emerged as core priorities. For the first time, popular science publicity was introduced under demand-side tools, signifying a policy orientation toward stimulating public demand. During this phase, policy attention to healthcare providers and patients continued to rise, reflecting a transition from emphasizing system construction to focusing on the capabilities of service suppliers and the experience of end beneficiaries.

## 4 Discussion

### 4.1 Outcomes

In this study, we established an analytical framework encompassing tool typology, thematic salience, and stakeholder configurations, based on Rothwell and Zegveld’s policy tool theory. Key insights were derived through bibliometric and content analyses of 41 rehabilitation medical service policies issued in mainland China over a 13-year period. The findings revealed that China’s rehabilitation medical service policy framework demonstrates institutional completeness, characterized by well-defined objectives, multifaceted implementation modalities, and favorable developmental ecosystems.

Environmental policy tools are predominantly utilized, with the “strategic road-mapping” subcategory receiving considerably high emphasis compared to other components within the same category. In contrast, both the supply-side and demand-side policy tools exhibited suboptimal deployment levels and notable internal structural imbalances. These findings align with those of a previous study (34).

Thematic analysis revealed that China’s rehabilitative healthcare system remains in an early developmental phase across both technological and institutional dimensions. Current strategic priorities are primarily concentrated in institutional rehabilitation frameworks, community-based intervention models, and Traditional Chinese Medicine (TCM) rehabilitation paradigms. Lexical frequency mapping identified psychotherapeutic interventions and TCM-based rehabilitation as pivotal developmental vectors. Notably, the ongoing structural integration of nursing support systems with rehabilitative protocols underscores the need for specialized therapeutic competencies in service delivery.

Based on stakeholder and cross-analysis results, provider organizations (42.95%) emerged as the core target of policies, followed by regulatory authorities (19.13%) and service recipients (19.80%). Clinical practitioners and the rehabilitation capital market each accounted for only 9.06%. The policy content directed at these latter groups is predominantly passive and response-oriented (e.g., skills training for medical staff or equipment configuration requirements for the capital market). In contrast, proactive and incentive-based clauses (e.g., career development support for medical staff or innovation subsidies for the capital market) are largely absent. This reflects a structural pattern characterized by a “focus on core entities and absence of vulnerable entities.”

### 4.2 Underlying reasons

Analyzing from the triangular dimension of “Outcome–Subject–Governance,” the observed imbalance in policy tool use and stakeholder representation may stem from adaptive responses corresponding to the development stage of rehabilitation services in China. From 2010 to 2023, China’s rehabilitation medicine sector was in an early, developmental phase, essentially “starting from scratch.” Guided by the incremental decision-making theory (35), the government prioritized the use of environmental policy tools (58.05%) to rapidly establish a policy framework. These tools are characterized by broad coverage, low cost, and high flexibility, and may guide development directions through strategic road-mapping (33.89%). In contrast, supply-side tools (32.98%) require long-term resource investment, whereas demand-side tools (9.06%) rely on multi-stakeholder collaboration. Both were challenging to implement on a large scale in the early development phase, leading to a stage-specific imbalance in policy tool application.

Second, the uneven representation of stakeholders may stem from significant disparities in their power positions and participation levels throughout the policy process. Drawing on the “Ladder of Citizen Participation” theory (36), government agencies and healthcare institutions occupy the “citizen power” tier at the top of the ladder, serving as policy formulators and core implementers, respectively. Cross-analysis of policy texts reveals that environmental tools are largely directed toward these two entities (48 entries for the government and 82 entries for healthcare institutions), which confirms their dominant status. Patients reside in the “symbolic participation” tier in the middle of the ladder: while they are beneficiaries of policies, they are not endowed with substantive authority to influence decision-making. Rehabilitation medical staff and the rehabilitation capital market occupy an even lower tier at the bottom of the ladder, functioning as passive respondents who are merely informed unilaterally. Relevant policies mostly consist of requirements imposed on them rather than provisions to motivate their initiative, and their core demands lack institutionalized channels for expression and influence.

Finally, limitations within the governance structure have constrained both the optimization of policy tools and collaboration among stakeholders. Influenced by the “administrative-led” tradition in China’s healthcare sector (37, 38), rehabilitation medical services have developed a “government–medical institution” dual governance model. A total of 48 “environmental tools–government” codes and 82 “environmental tools–medical institution” codes constitute the core policy chain, while collaborative participation mechanisms for medical staff, capital, and the public are absent (e.g., no policies mention medical staff participation in policy revision). This governance structure not only limits the efficiency of resource allocation in supply-side tools but also makes it difficult for demand-side tools to stimulate market and public demand. As a result, it reinforces existing patterns of tool imbalance and stakeholder differences.

Compared with developed countries, the Chinese rehabilitation policy model identified in this study presents distinct characteristics. The rehabilitation policy systems in European and American countries have become relatively mature through long-term development, driven by a robust market mechanism, diversified insurance payment schemes, active industry associations, and patient organizations in a collaborative manner. China’s model, characterized by “dominance of environmental tools and a dual core of government-medical institutions,” represents a typical “top-down” system construction path. This reflects the strategic choice of a developing country like China to rapidly establish a policy framework “from scratch” during a specific stage of development.

### 4.3 Recommendations

To address the prominent issue that demand-side tools account for only 9.06%, priority measures can be advanced in the later stage of the 14th Five-Year Plan. For instance, a rehabilitation service voucher system should be established: issue universal rehabilitation service vouchers to key groups such as post-operative patients and older adults, clarify the voucher amount, scope of use, and redemption procedures. By directly subsidizing the demand side, this system reduces the threshold for rehabilitation consumption, guides the public to actively use rehabilitation services, and converts potential demand into effective demand. Additionally, the coverage of rehabilitation science popularization can be expanded: collaborate with medical institutions, communities, and new media platforms to develop a rehabilitation knowledge popularization program, design differentiated science content for different groups, and enhance public awareness of rehabilitation through public lectures, community free clinics, and other forms—thereby driving rehabilitation service consumption from the source of demand.

Focusing on the core dimensions of supply-side tools such as resource allocation and disciplinary development, two aspects of work can be promoted. First, the rehabilitation resource allocation mechanism can be optimized: centering on hierarchical medical system, establish a connection system of “upper-level hospital assessment + community rehabilitation training + home-based rehabilitation guidance,” and use special fiscal funds to subsidize the update of primary-level rehabilitation equipment. Second, a special program for rehabilitation talent training should be implemented: conduct regular training on rehabilitation skills for existing medical staff, set up special allowances for rehabilitation talents, and attract talents to flow to primary-level and remote areas.

To tackle the problems of excessively high proportion of goal planning and insufficient implementation in environmental tools, refinements can be made in two aspects. First, an assessment mechanism for rehabilitation policies could be established: incorporate indicators such as rehabilitation service coverage into the health work assessment system of local governments, and conduct regular evaluations of policy implementation effects. Second, the coordination of medical insurance reimbursement policies can be strengthened: further expand the coverage of rehabilitation programs under medical insurance, explore pilot medical insurance payment for new service models such as home-based rehabilitation and tele-rehabilitation. Leveraging the medical insurance lever to drive the development of the rehabilitation service market will also provide institutional support for the implementation of demand-side and supply-side tools.

### 4.4 Limitations

The transferability of the research findings to other contexts may be limited. Additionally, potential bias from researcher subjectivity may influence data interpretation, although this was mitigated through interaction checks within the research team.

### 4.5 Conclusion

From the perspective of policy tools, this study reveals the structural imbalance of China’s rehabilitation policies—characterized by “the dominance of environmental tools and insufficient supply-side and demand-side tools”—as well as the governance feature of “government-medical institution” as the core with insufficient participation of other stakeholders. Compared with international rehabilitation and nursing policies, China’s policy framework still needs to be strengthened in terms of the improvement of service systems and the diversification of tool utilization. This study provides a new perspective for international policy research.

## Data Availability

The original contributions presented in the study are included in the article/supplementary material, further inquiries can be directed to the corresponding author.
